# The Mayer-Rokitansky-Küster-Hauser syndrome (congenital absence of uterus and vagina) – phenotypic manifestations and genetic approaches

**DOI:** 10.1186/1477-5751-5-1

**Published:** 2006-01-27

**Authors:** Daniel Guerrier, Thomas Mouchel, Laurent Pasquier, Isabelle Pellerin

**Affiliations:** 1 CNRS UMR 6061, Génétique et Développement, Université de Rennes 1, Groupe IPD, IFR140 GFAS, Faculté de Médecine, Rennes, France; 2 Unité de Génétique Médicale, Hôpital Sud, Rennes, France; 3 Service de Gynécologie Obstétrique, CHU de Rennes, Rennes, France

## Abstract

The Mayer-Rokitansky-Küster-Hauser (MRKH) syndrome affects at least 1 out of 4500 women and has for a long time been considered as a sporadic anomaly. Congenital absence of upper vagina and uterus is the prime feature of the disease which, in addition, is often found associated with unilateral renal agenesis or adysplasia as well as skeletal malformations (MURCS association). The phenotypic manifestations of MRKH overlap various other syndromes or associations and thus require accurate delineation. Since MRKH manifests itself in males, the term GRES syndrome (Genital, Renal, Ear, Skeletal) might be more appropriate when applied to both sexes. The MRKH syndrome, when described in familial aggregates, seems to be transmitted as an autosomal dominant trait with an incomplete degree of penetrance and variable expressivity. This suggests the involvement of either mutations in a major developmental gene or a limited chromosomal deletion. Until recently progress in understanding the genetics of MRKH syndrome has been slow, however, now HOX genes have been shown to play key roles in body patterning and organogenesis, and in particular during genital tract development. Expression and/or function defects of one or several HOX genes may account for this syndrome.

## Introduction

Müllerian and Wolffian ducts are the primordia for the internal reproductive systems of females and males respectively and co-exist in the undifferentiated embryo until genetic sex triggers differentiation of either ovaries or testes. Müllerian ducts differentiate into Fallopian tubes, uterus, cervix and upper part of the vagina while the Wolffian ducts degenerate. In the male testicular production of anti-Müllerian hormone (AMH) and androgens leads to the development of the Wolffian ducts into vas deferens and seminal vesicles.

Various forms of Müllerian abnormalities range from minor anatomical variations up to total aplasia, the latter being diagnosed as Mayer-Rokitansky-Küster-Hauser (MRKH) syndrome in 90% of affected women. The frequency of congenital absence of vagina and uterus is not yet entirely clear, although reported incidences vary from 1 in 4,000 to 5,000 female births [1,3]. In women presenting with primary amenorrhea, MRKH is fairly common, being second to gonadal dysgenesis as a cause of amenorrhea [4,5]. Although the vast majority of cases of MRKH seem to be sporadic [5] familial aggregates have been reported [1,6,8].

Women with Müllerian agenesis show normal 46, XX karyotypes [9-12], normal external genitalia and functional ovaries [13,14], there are however cases of polycystic ovaries [15,16] and ovarian tumors [17-20]. Examination reveals an absence or severe hypoplasia of the upper vagina as well as frequent uterine agenesis. Due to a different embryonic origin, the lower third of the vagina is always present, [21,22]. There appears to be two subtypes of MRKH: the typical (also called type I or isolated) and the atypical form (type II): the frequency of type II being much greater [23]. The typical form is characterized by laparoscopic or laparotomic findings of symmetric muscular buds (the Müllerian remnants) and normal Fallopian tubes; this is referred to as the so-called Rokitansky sequence, where only the caudal part of the Müllerian duct (upper vagina and uterus) is affected [24] (OMIM 277000 [25]). The atypical form shows, in addition, asymmetric hypoplasia of one or two buds, with or without dysplasia of the Fallopian tubes: this is often associated with other anomalies, including mainly renal defects (unilateral agenesis or ectopia of one or both kidneys, horseshoe kidney, in about 40–60% of patients) [26], cervico-thoracic (asymmetric, fused or wedged vertebrae, scoliosis and Klippel-Feil anomaly in about 20% of patients) [26] and, to a minor extend, hearing defects [27,28] and digital anomalies of varying severities [1,10,29-33]. For these reasons, clinicians use the term MURCS (MÜllerian Renal Cervical Somite) association [12,34] which is the most severe form of the disorder (OMIM 148860 and 601076 [25]). It may be attributed to an alteration of the blastema of the cervicothoracic somites and the pronephric ducts which, by the end of the fourth week of fetal life, have an ultimately spatial relationship [12]. These overall features clearly differentiate the MRKH syndrome from other defects of genital tract development such as androgen peripheral insensitivity (patients 46, XY) [6,35] or Turner's syndrome (patients 45, X) [36].

### Treatment

Treatment is usually delayed until the patient is ready to start sexual activity. It may be either surgical or non-surgical but the chosen method needs to be tailored to the individual needs and motivation of the patient and the options available [37]. For instance the Vecchietti operation is a mixture of surgical and non-surgical methods of creating a neovagina and has been performed frequently in Europe over the last 20 years [38].

In addition, it is important to manage psychological symptoms in women with Müllerian agenesis. Indeed a young woman who discovers that she has a congenital malformation involving her reproductive organs may develop extreme anxiety about her feminity and physical image. The psychological adjustment and general attitude are therefore also very important in deciding what procedure should be used and when [39].

### Similarities of MURCS association with other syndromes (Table 1)

**Table 1:** Genetic diseases featured by Müllerian, renal and skeletal malformations: similarities and differences with MRKH syndrome/MURCS association.

**MRKH/MURCS**	**Klippel-Feil**	**VATER**	**FAV spectrum**	**Winter synd.**	**HRA**
OMIM 277000/601076	OMIM 148860	OMIM 192350	OMIM 164210	OMIM 267400	OMIM 191830
**uterus aplasia**		*uterus aplasia*	uterus aplasia		uterus malform.
**vaginal aplasia**		vag. *aplasia *atresia (1)	vaginal aplasia	**vaginal atresia (1)**	vaginal malform.
**kidney malform. (2)**	kidney malform. (2)	**kidney malform. (2)**	kidney malform. (2)	**renal agenesis (3)**	**renal agenesis (3)**
**short neck**	**short/web neck**				
**scoliosis**	**scoliosis**				
**other vert. defects**	**other vert. defects**	**other vert. defects**	**other vert. defects**		
rib defects	rib defects	rib defects			
thumb hypoplasia		**thumb hypoplasia**	**thumb hypoplasia**		
		**radius hypoplasia**			
		**polydactyly**			
**deafness**	deafness		**deafness**	**deafness**	
*malf. ext. ears*		malf. ext. ears	**malf. ext. ears**		
			low set ears		
facial asymmetry	**facial asymmetry**		**facial asymmetry**		
			**malar hypoplasia**		
			**maxilary hypoplas.**		
*micrognathia*			**micrognathia**		
			**macrostomia**		
			strabismus		
			microphtalmos		
			iris coloboma		
			glaucoma		
cleft lip/palate	cleft palate w/o lip		cleft lip/palate		
			**cleft tongue**		
		**single ombilic artery**			
		**anal atresia**			
		**trach-oesoph. fistula**			
*cardiac defects*	cardiac defects	**cardiac defects**	cardiac defects		

Frequency of malformations is indicated as follow: **very frequent **occasional *rarely observed*

(1): vaginal atresia affects the lower part of the vagina and is far different from vaginal aplasia found in MRKH/MURCS and which affects the upper (~2/3) part of the vagina.

(2): kidney malformations include unilateral agenesis, ectopia of one or both kidneys, horseshoe kidney. Bilateral agenesis can be found *post mortem*.

(3): unilateral or bilateral renal agenesis.

Although utero-vaginal aplasia should be the obligatory start point in establishing MRKH syndrome, associated malformations (see introduction) may be misleading and steer clinicians towards a false diagnosis. On the other hand, finding of Müllerian abnormalities in a patient showing features of an other predominant syndrome can also be confusing. Therefore, the use of the general term "Müllerian abnormalities/anomalies" should be banished as it refers to variable malformations ranging from minor anatomical variations up to total aplasia [21,30]. In addition, accurate delineation of associated malformations may be a great help in establishing a MRKH syndrome [22].

Indeed, most of the defects that form parts of the MURCS association [12] can be observed optionally and in various combinations as do those described in the VATER association (Vertebral defects, Anal atresia, TracheoEsophageal fistula and/or oesophageal atresia, Radial dysplasia, Renal defects) [40,42], both syndromes sharing in common several of these defects, mainly renal, vertebral and upper limb but being distinct clinical entities [12,30,43]. Although frequency of renal defects is about the same in both syndromes [1,44], utero-vaginal dysplasia, the prime feature of the MURCS association, is however a rare trait of VATER patients. Finally, discrepancy can be made upon features not found in the MURCS but frequent in VATER, such as T-E fistula with oesophageal atresia (70%) and anal atresia (80%) [43,45].

Another source of confusion is that of the Winter syndrome [46], commonly described in the literature as a "syndrome of renal, genital and middle ears anomalies" [46-49]. Patients are initially found to have bilateral stenosis of the external auditory canals in their youth but diagnosis is made only after primary amenorrhoea has led to vaginal atresia and unilateral renal agenesis being revealed after further examination [49]. In the reports cited above, the genital anomalies are however restricted to a distal vaginal atresia which is far different from the Müllerian aplasia observed in the MRKH syndrome, even in its mildest form, where only the upper vagina is absent. Moreover, while partial or total Müllerian aplasia confers irreversible sterility, vaginal atresia can be surgically corrected to permit pregnancy [49].

MRKH has also been described in association with other syndromes, especially hereditary renal adysplasia (HRA) [50,53] and facio-auriculo-vertebral (Goldenhar) spectrum (FAVS) [54,58] in women otherwise presenting normal karyotypes and normal female secondary sex characteristics. In HRA, where uni- and bi-lateral renal agenesis (URA and BRA) can be observed in the same family, Müllerian anomalies are rarely encountered and appear to be an occasional secondary manifestation of the syndrome. They consist in minor abnormalities such as didelphic uterus [50] or unicornate uterus [51] that can often be surgically corrected to permit pregnancy. Finally, none of the other malformations that form part of the MURCS association, such as vertebral or auditory defects, is ever found in HRA [50,53]. Investigation of family history, when available, should then allow a distinction to be made, according to whether URA and/or BRA is the only malformation seen in the family (familial HRA) or if other anomalies specific to MRKH, such as vertebral or isolated Müllerian aplasia are evidenced.

Concurrence of MURCS and FAVS is puzzling. Indeed some reports on MURCS include all combinations of ear, vertebral and facial defects that are the main features of FAVS. This consequently leads to question whether MURCS and FAVS are two separate entities or various manifestations of the same syndrome/association. Most of the reports are in favour of independent malformative associations as the vast majority of each MURCS or FAVS occurs without any feature of the other (see Table 1).

### Genetics of the MRKH syndrome

Although the pathogenesis of Müllerian aplasia with or without associated malformations is now well described, its etiology remains unknown [59] and therefore, its familial occurrence is of considerable interest. The apparent lack of familial transmission initially suggested the involvement of non-genetic factors [21] such as thalidomide-like teratogens [12,60] however pregnancy histories, when available, have never revealed any association with any record of drug use, illness, or exposure to known teratogens.

In almost all reports, karyotypes of patients are that of normal 46, XX women. Rare chromosome abnormalities have been found associated with Müllerian aplasia, such as mosaicisms [61,62], rearrangements/deletions [11], often associated with gonadal dysgenesis [63,65], or both [66]. It is noteworthy that, in these rare cases, only the X chromosome seems to be involved and therefore appears to carry a gene(s) involved in early differentiation of at least both gonads and Müllerian ducts.

Most reports of Müllerian dysplasia/aplasia have focused on patients' defects and their surgical treatment, without reporting family histories. This may explain why the majority of cases have been assumed to be sporadic. However, reference work of Griffin and co-workers [1] showed significant incidence (>20%) of familial cases: they described a triad of main features of MRKH – Müllerian aplasia, spine and kidney involvement, these two latter being optionally observed in combination with the first and interestingly, occurring in more distant relatives as well as mothers. This has been confirmed by other reports [7,53,67] and raised the question of whether or not the MRKH was manifested in the male. In fact combinations of Wolffian duct agenesis or severe hypoplasia, with or without renal and/or skeletal anomalies have been described, as both congenital unilateral renal agenesis associated with ipsilateral agenesis of the vas deferens [68,69], and as primary infertility due to azoospermia associated with Klippel-Feil deformity [70] or segmentation abnormalities of the cervico-thoracic spine and hearing impairment [71,72]. In azoospermic patients, the infertility seems to be attributable to uni- or bi-lateral defect of vas deferens development ranging from hypoplasia [70] to agenesis [69,72,73], leading to so-called obstructive azoospermia. Given that the acronym MURCS cannot apply to males, it has been suggested that the male counterpart ARCS (Azoospermia, Renal anomalies, Cervicothoracic Spine dysplasia) would be more suitable to designate this condition [71,72] although GRES (Genital Renal Ear Skeletal) which applies to both sexes [23] would be even more appropriate. As therefore expected, cases exist where both MURCS and ARCS have been found in the same family [73].

From the data cited above, it seems that Müllerian aplasia can represent only one manifestation of a variably expressed genetic defect, and since the detection and definition of the features of MRKH have an element of unreliability, it is likely that the real frequency of familial occurrence has been underestimated. Furthermore, a contribution to the genetic disorder can clearly be transmitted by either father or mother, neither of whom may express any of the defects or at least those which are not deleterious for reproduction (for example unilateral renal agenesis, skeletal anomalies or deafness). Consequently, although these overall data are broadly compatible with a classic model of multifactorial/polygenic inheritance, as often suggested [5,8,21,74,75], it seems that a more likely mode of transmission is that of an autosomal dominant trait with an incomplete degree of penetrance coupled with a highly variable expressivity of a single mutant gene as previously hypothesized [1,53,73] or of a limited deletion undetectable in standard karyotypes.

### Candidate gene approaches

Up to now, the lack of families with informative genetic histories has not allowed the identification of any locus by standard genetic linkage analysis. An alternative approach based either on an apparent association with other genetic diseases or on pleiotropic action during embryogenesis and consequent manifestations as developmental field defects, has led to several genes investigation. Genetic association of MRKH with galactosemia [76] or with cystic fibrosis [4] has been analysed but neither the gene for galactose-1-phosphate uridyl transferase (GALT) [77] nor the gene encoding the CFTR chloride channel [4] showed any mutation and/or polymorphism associated with Müllerian aplasia. Genes acting during early development such as WT1 and PAX2 have also been suggested as candidates, although so far with no demonstration of their role in MRKH syndrome [78,79]. Moreover, anti-Müllerian hormone or its receptor, together responsible for Müllerian duct regression in male fetuses [80] and therefore good candidates, have no etiological role in the syndrome [81]. It is noteworthy that mutations within the hepatocyte nuclear factor (HNF)-1β gene, originally found associated with MODY-type diabetes [82] and with diabetes mellitus, renal dysfunction and genital malformations [83], were suspected to account for an MRKH-like phenotype [83,84] (OMIM 158330 [25]). However, absence of phenotype/genotype correlation in addition to non-MRKH uterine malformations [84] is not consistent with a straight relationship between MRKH syndrome and HNF-1β gene defects. Finally, we investigated a patient showing uterovaginal agenesis combined with unilateral renal aplasia and MODY-type diabetes and did not find any mutation in the HNF-1β gene (unpublished results).

### The particular case of Wnt genes

Wnt genes encode secreted glycoproteins that regulate cell and tissue growth and differentiation during embryogenesis [85]. Three members of the Wnt gene family, Wnt4, Wnt5a and Wnt7a, are expressed at high levels in the developing female genital tract, in the mouse model [86]. Homozygotic inactivation of each of these genes results, in all cases, in severe Müllerian ducts defects, ranging from DES exposure-like malformation [87] to total failure of Müllerian duct formation amongst numerous lethal defects at birth [88]. A loss-of-function mutation in the Wnt4 gene was recently described in association with an absence of Müllerian-derived structures, unilateral renal agenesis, and clinical signs of androgen excess in an 18-year-old woman [89]. The congenital malformations observed in this patient suggested a MRKH-like phenotype although no skeletal anomaly was evidenced. However the complete absence of Müllerian structure seems to be due to ectopic expression of AMH by masculinized ovaries during fetal life, as explained by the authors. In addition, ovarian masculinization is never observed in well diagnosed MRKH syndrome and therefore the loss-of-function mutation of Wnt4 is not a very likely the cause of the syndrome. Sequencing of the Wnt4 gene in 19 MRKH patients [90] supports this idea. Wnt4 is a major developmental gene required for normal kidney and female gonad differentiation and its role takes place early in embryogenesis, prior to Müllerian differentiation [88]. It may, in addition, play an important role in Müllerian duct differentiation. If so, Müllerian duct-targeted disruption of the Wnt4 gene in the mouse may give an answer. For instance, the role of Bmpr1 in Müllerian duct regression was proven using this tissue-specific targeting strategy [91].

The involvement of Wnt5a and Wnt7a during female genital tract development has been demonstrated [92,93]: the female genital tract malformations in Wnt5a and Wnt7a mutant mice are similar to those described in HOXA11 and HOXA13 transgenic mice bearing homozygotic null alleles [94,95]. Interestingly, uterine malformations observed in these latter knocked-out mice strongly resemble to those reported for animals exposed in *utero *to DES and in which HOXA10, HOXA11 [96] as well as Wnt7a gene expression is altered [87].

It therefore appears that some Wnt and HOX genes products co-operate to pattern female genital tract during mouse embryogenesis as recently postulated [92]. Data discussed above tend to show that Wnt4, Wnt5A and Wnt7A genes are not good candidates for genes contributing to MRKH syndrome: however this generalization does not apply to the HOX genes.

### The HOX genes hypothesis

In 1999, JL Simpson [21] deplored the absence of molecular progress "*despite potentially attractive candidate genes*" – he also pointed out that "*a great interest lies in developmental genes, like those in the HOX series*". The point has been made again recently [59,97]. Indeed there are clearly very few genes known to be involved in the differentiation of Müllerian duct as well as kidney and skeleton: these are mainly the HOX genes which have been studied through their pattern of segmentally based expression and by *in vivo *inactivation (knock-out) in the mouse model. It emerges from these experiments that HOXA10 [98], HOXA11 [94] and HOXA13 [95] seem to be required for correct Müllerian duct differentiation. Furthermore, these latter genes are also expressed in the developing kidney [99] and patterning of the skeleton requires both HOXA10 and HOXA11 [100].

In human, mutations within HOXA13 gene or deletion of the HOXA gene cluster mainly affect uro-genital tract and skeleton. Mutations in HOXA13 coding region cause handfoot-genital syndrome (HFGS) which is characterized by hand malformations, hypospadias in males, Müllerian duct fusion defects in females (ranging from longitudinal vaginal septum to double uterus with double cervix) and urinary tract malformations in both sexes [101,102]. Surprisingly, deletion of the whole HOXA cluster does not cause more uro-genital anomalies than single mono-allelic HOXA13 mutations [103]. This suggests that either mono-allelic dominant mutations within HOXA9, -A10 or -A11 may account for the MRKH syndrome or this can be due to other mechanisms such as mis-regulation of HOXA genes, affecting either transcription rate or spatio-temporal expression: the recent finding of a mutation within the HOXA13 gene promoter [104] strengthens this hypothesis.

## Conclusion

Incidence of the MRKH syndrome/MURCS association has probably been underevaluated mainly because it has, until recently, been seen as a female-specific and sporadic disorder. Isolated features of the triad of main malformations, including kidney agenesis and/or skeletal defects, were consequently not investigated in all probands' relatives, including males who can also be affected. This is understandable given the incomplete degree of penetrance, variable expressivity and similarities of this syndrome with other genetic disorders.

Treatment which consists in creating a neovagina is generally offered to patients when they are ready to start sexual activity. Moreover, everyday improvement of medical technologies allows, in many countries, women to appeal for in *vitro *fertilization and surrogate pregnancy to bypass the absence of inner genital tract. The number of such women will probably increase with time. This is why characterization of the genetic events responsible for this syndrome is of major importance.

## Authors' contributions

- DG initiated the study in IP's group and has been leading this research program since then.

- TM co-initiated this program and delineated MRKH syndromes

- LP contributed to the diagnosis and was in charge of medical genetics

- IP created a new research group focused on molecular events triggering normal and pathological differentiation of the Müllerian ducts. She therefore offered the opportunity to DG to set up a proper clinical research program aiming at understanding the genetics of MRKH syndrome.
